# Background visual motion affects responses of an insect motion‐sensitive neuron to objects deviating from a collision course

**DOI:** 10.14814/phy2.12801

**Published:** 2016-05-20

**Authors:** Jasmine M. Yakubowski, Glyn A. McMillan, John R. Gray

**Affiliations:** ^1^ Department of Biology, 112 Science PlaceUniversity of SaskatchewanSaskatoonSKCanada

**Keywords:** DCMD, locust, motion detection, optic flow, vision

## Abstract

Stimulus complexity affects the response of looming sensitive neurons in a variety of animal taxa. The Lobula Giant Movement Detector/Descending Contralateral Movement Detector (LGMD/DCMD) pathway is well‐characterized in the locust visual system. It responds to simple objects approaching on a direct collision course (i.e., looming) as well as complex motion defined by changes in stimulus velocity, trajectory, and transitions, all of which are affected by the presence or absence of background visual motion. In this study, we focused on DCMD responses to objects transitioning away from a collision course, which emulates a successful locust avoidance behavior. We presented each of 20 locusts with a sequence of complex three‐dimensional visual stimuli in simple, scattered, and progressive flow field backgrounds while simultaneously recording DCMD activity extracellularly. DCMD responses to looming stimuli were generally characteristic irrespective of stimulus background. However, changing background complexity affected, peak firing rates, peak time, and caused changes in peak rise and fall phases. The DCMD response to complex object motion also varied with the azimuthal approach angle and the dynamics of object edge expansion. These data fit with an existing correlational model that relates expansion properties to firing rate modulation during trajectory changes.

## Introduction

Natural environments contain an array of complex sensory stimuli. Animals must extract behaviorally relevant sensory cues from their surroundings in order to elicit an appropriate avoidance response. Therefore, sensory signals occurring within important temporal and spatial windows are integrated with proficiency (Leo and Noppeney 2014). Detection of distinct sensory signals is subject to perceptual quality and is also affected by the type of visual environment an organism is exposed to. While stationary animals may be able to clearly detect salient visual stimuli, more complex visual environments, such as those generated by optic flow resulting from self motion, present challenges that could make detection more difficult.

Migratory locusts (*Locusta migratoria)* are well known for swarming at very high population densities, which can reach up to 1000 individuals/m^3^ (Uvarov 1977). Within a swarm, conspecifics or predators may approach the locust from many different angles. Therefore, a locust's natural environment is comprised of complex combinations of visual stimuli (produced by self‐motion or object motion) that may be translating, receding, or looming (Uvarov 1977). These animals must be able to detect such visual stimuli in order to avoid collision or capture. Looming objects provide critical visual information regarding an impending collision or an approaching predator or conspecific (Gabbiani et al. 1999) and have been shown to evoke collision avoidance behaviors in locusts (Gray et al. 2001; Santer et al. 2005; Simmons et al. 2010; Chan and Gabbiani 2013; McMillan et al. 2013).

*L. migratoria* is an established neuroethological system for studying collision avoidance behavior and visual processing. The Lobula Giant Movement Detector (LGMD) and the Descending Contralateral Movement Detector (DCMD) make up a visual processing pathway preferentially responsive to objects approaching on a direct collision course at a constant velocity (i.e., looming) (Schlotterer 1977; Rind 1984; Rind and Simmons 1992) that functions throughout the life of the locust (Simmons et al. 2013). The LGMD synapses onto the DCMD in a one‐to‐one spike ratio (O'Shea and Williams 1974; Rind 1984) and is often referred to as an angular threshold detector since the number of spikes produced by the LGMD is directly related to the subtense angle of an approaching object (Gabbiani et al. 1999). The DCMD descends through the ventral nerve cord and makes excitatory connections with motor neurons involved in flight steering (Simmons 1980). Extensive research has been conducted on the involvement of the LGMD/DCMD pathway in looming detection as well as its potential role in escape behaviors (Rind and Simmons 1992; Gray et al. 2001; Santer et al. 2006, 2012; McMillan and Gray 2012).

Although the DCMD responds preferentially to looming objects, it also responds to complex object motion (Rind and Simmons 1992). For example, modulation of DCMD activity reflects trajectory changes when an object transitions to or away from a collision course (McMillan and Gray 2012). These responses are further modulated by the object's velocity (Dick and Gray 2014) and changes in background visual flow (Silva et al. 2015). However, no studies have addressed how this visual pathway responds to compound trajectories that transition away from the animal in complex visual environments. To further address the question on how complex visual information is encoded, we recorded DCMD activity in response to objects that transition away from a collision course in the presence or absence of background motion. DCMD response parameters varied depending on trajectory and background types. We found that background complexity affects the magnitude of DCMD response parameters to stimuli that transition away from a collision course, and in some cases, even removes the response completely. These data show that the modulation of the DCMD firing rate reflects aspects of this type of complex visual motion in addition to those previously studied (McMillan and Gray 2012; Dick and Gray 2014; Silva et al. 2015). Overall, responses varied depending on the type of trajectory and background.

## Materials and Methods

### Animals

Experiments were conducted using adult *L. migratoria* acquired from a crowded colony maintained at the University of Saskatchewan in Saskatoon, SK, Canada. We used 20 adults (18 males and 2 females) that were at least 3 weeks past the imaginal molt. There were no significant differences in DCMD firing parameters for control looms between males and females (data not shown). Locusts were reared with a 12:12 h light:dark cycle at 25–28°C and experiments were performed at room temperature (~25°C) at approximately the same time of the animals’ light cycle (mid‐afternoon).

### Preparation

The legs and wings were removed before a rigid tether was attached to the ventral surface of the thorax using 3M^™^ Vetbond^™^ Tissue Adhesive 1469SB (3M Animal Care Products, St. Paul, MN). A patch of ventral cervical cuticle was excised to expose the underlying pair of ventral nerve cord connectives anterior to the prothoracic ganglion. Exposed tissue was bathed in locust saline (147 mmol NaCl, 10 mmol KCl, 4 mmol CaCl_2_, 3 mmol NaOH, 10 mmol Hepes, pH 7.2). The preparation was transferred to a recording stage, where a single hook silver electrode was positioned under the right or left ventral nerve cord. The recording site was insulated with a Vaseline and mineral oil mixture once high signal:noise neural responses were observed. Neuronal recordings were taken from the ventral nerve cord opposite to the side of stimulus presentation, since the main DCMD axon descends contralateral to the eye being stimulated (Rind 1984). A copper ground wire was also placed directly into the abdomen of the locust. The locust was orientated dorsal side up and aligned in both azimuthal and elevation planes 12 cm from the apex of a rear projection dome screen. In this setup, 0**°** was directly in front of the locust and 90**°** was designated as the azimuthal center of the eye used in experimentation, regardless of the side of the animal. The preparation was left to acclimate for ~20 min before commencing experimentation and ~10 min in front of each background before visual stimuli were presented. We maintained 3‐min intervals between presentations to prevent neural habituation.

### Visual stimuli

Visual stimuli were generated using Vision Egg visual stimulus generation software (Straw 2008). Approach parameters of a looming object can be described by a ratio of the half size of the object (*l*) divided by the absolute velocity during approach |*v*| (Gabbiani et al. 1999). A black 7‐cm disk travelling at 300 cm/sec (*l/|v| *= 12 msec), scaled in real time at 85 frames/sec, was used to produce object motion. Stimuli were projected onto a rear projection dome screen with an InFocus DepthQ LCD projector. The projections were coded within Vision Egg to account for the curvature of the dome screen. We used a 1.2 msec TTL pulse included in each video frame along with the Vsync pulse from the video card (NVIDIA GeForce 7900 GTX 512 MB) to align neuronal recordings with visual stimuli. The Michelson contrast ratio (0.49) and luminance values were similar to those used in previous studies (Guest and Gray 2006; McMillan and Gray 2012; Dick and Gray 2014; Silva et al. 2015).

**Figure 1:**
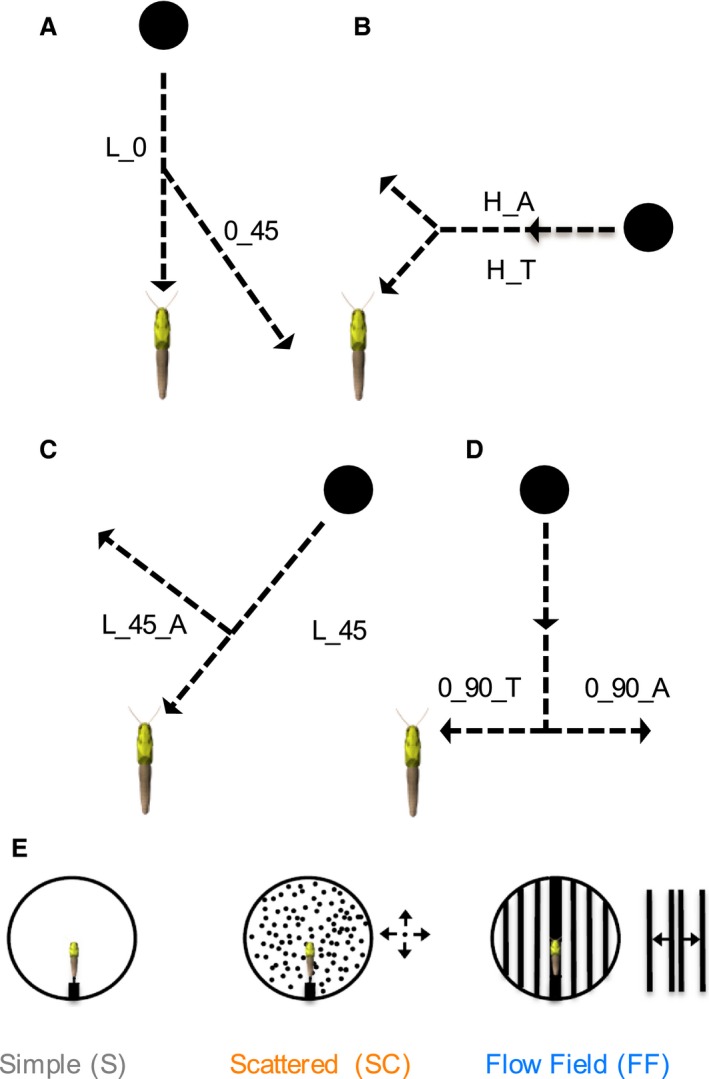
Computer generated visual stimuli. Black disks (7 cm) travelling at a velocity of 300 cm/sec were presented to the locust along eight different trajectories, each against three different visual backgrounds. Three types of object motion were presented: looming, transitions to looming, and transitions away from looming. (A) L_0 was designated as a frontal loom towards the animal. A transition from this trajectory at a 45**°** angle occurred at 60 cm away from the animal (0_45). (B) Disk approaching orthogonal to the long axis of the locust's body 50 cm anterior to the eyes, that transitioned toward (H_T) or away (H_A) from looming at an angle of 45**°** starting 75 cm away from the locust. (C) Disk travelling along a looming trajectory at a 45**°** angle to the locust's eye (L_45). The disk also transitioned 43 cm from the eye 90**°** orthogonal to the original trajectory (L_45_A). (D) Disk motion started parallel to the long axis of the locust's body in the anterior field of view, offset from locust's long axis by 30 cm, approached until parallel with the middle of the locust's eye (90°) and transitioned toward (0_90_T) or away from (0_90_A) the locust. (E) Visual representations of the three stimulus backgrounds used: Simple (S), Scattered (SC), or Flow field (FF). Modified from Silva et al. (2015). Arrows in A–D represent the relative direction of object motion. Arrows next to diagrams in E indicate that the objects moved in all directions (scattered) or the vertical bars expanded outward from the center (flow field). See Materials and Methods for additional descriptions.

To examine DCMD responses to compound stimuli, particularly those transitioning away from a collision course, locusts were exposed to a randomized set of eight trajectories presented within three different visual backgrounds (Fig. 1). Visual backgrounds were also randomized for each animal. In addition, each locust was presented with a 0° (i.e., head on) loom presented at the start and end of each presentation series. All stimuli were presented within the azimuthal plane and at 0° elevation. Frontal looms (L_0) started from 400 cm away and loomed head‐on to the locust (0°); 0_45 transitioned away from the locust at a 45° angle relative to its initial path (looming at 0°), at a distance of 60 cm away from the locust (Fig. 1A). We define compound horizontal stimuli as traveling orthogonal to the long axis of the locust's body 50 cm anterior to the eyes, and transitioning toward (H_T) or away (H_A) from the locust at a 45° angle relative to their initial path at 75 cm away from the locust (Fig. 1B). L_45 loomed toward the locust at 45° and L_45_A transitioned away from looming at 43 cm from the locust 90° orthogonal to its initial 45° path (Fig. 1C). 0_90 trajectories started in front of the locust, offset from the longitudinal center by 30 cm, approached the locust until reaching 90° azimuth and then transitioned either toward (0_90_T) or away (0_90_A) from looming (Fig. 1D). All trajectories with a looming component subtended 32.5° of the locust's visual field at the end of the loom. Transition distances varied with trajectory type as values were chosen to provide a range of object expansion parameters to apply to our model (see below) and were based on previous experiments (McMillan and Gray 2012; Silva et al. 2015). Stimulus backgrounds were the same as those used in Silva et al. (2015) (Fig. 1E). The simple white background (S) contained no additional objects; the scattered background (SC) consisted of 600 black disks with an angular size of 4.6°, randomly moving along straight trajectories within a single plane orthogonal to the long axis of the locust at 2.8 cm/sec; and the flow field (FF) background was a modified vertical grating pattern comprised of vertical bars (angular size = 11.42° at the eye) moving in the azimuthal plane outwards from the apex of the projection dome at 13.8 cm/sec.

### Spike sorting and quantification of DCMD firing properties

**Figure 2:**
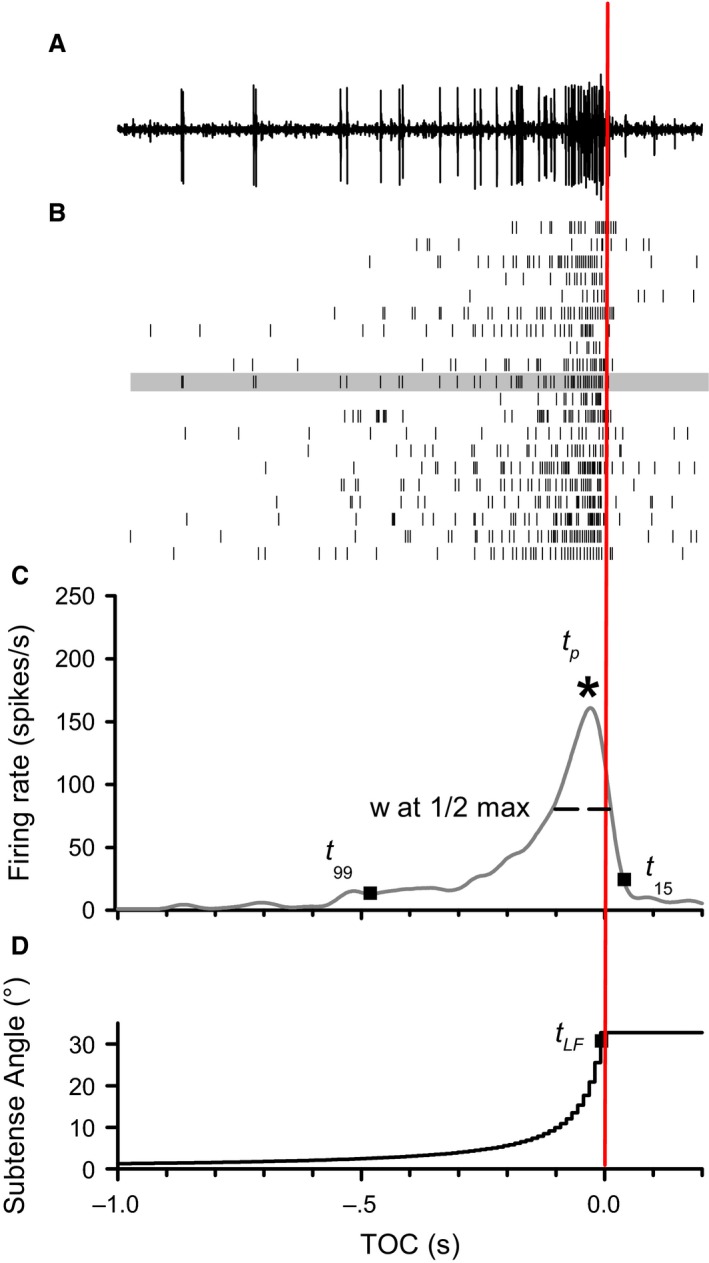
Responses to a frontal looming object against a simple background. (A) Individual raw neural recording from the left DCMD. (B) Raster plots of spike times during the approach (*n* = 20 locusts). Shaded rasters indicate spike times from the recording in A. (C) Mean DCMD peristimulus time histogram from all rasters in B. Labeled points on the DMCD response indicate the specific times used to calculate rise and fall phases. The start of the rise phase (*t*_99_) is when the firing rate exceeded a 99% confidence interval, and ended when the DCMD firing rate peaked (*t*_p_ *)*. The start of the fall phase was the time of the last frame of expansion (*t*_LF_ in D) and ended at the time of the nearest spike to 15% of the peak firing rate (*t*_15_). See Materials and Methods for a description of the calculation of TOT‐associated rise and fall phases (now shown). We also measured the number of spikes during approach, the amplitude and time, relative to TOC, of the peak firing rate (*), the duration of the peak at ½ peak amplitude (PW½M). See text for details on measurements associated with a time of transition. (D) The increasing visual subtense angle for a 7 cm black disk during a frontal loom. All panels time aligned to the projected time of collision (TOC, red vertical line).

For each stimulus presentation, we recorded neuronal activity, pulses time aligned with each frame of the stimulus, and vertical synchronization (Vsync) pulses from the video card. Neural activity was amplified with a differential AC amplifier (A‐M Systems, model No. 1700, Sequim, WA) with a gain of 10,000 and sampled at 25 kHz. To store the data, a RP2.1 enhanced real‐time processor (Tucker‐Davis Technologies, Alachua, FL) with Butterworth filter settings of 100 Hz (high pass) and 5 Hz (low pass) was used. The axon of the DCMD is the largest in the ventral nerve and produces characteristically large amplitude spikes (Fig. 2A), which were easily identified and isolated using threshold sorting in Offline Sorter (Plexon Inc., Dallas, TX). Spike times (Fig. 2B) were exported to Neuroexplorer (NEX Technologies, Littleton, MA) and used to create peristimulus time histograms (1‐msec bin) smoothed with a 50 msec Gaussian filter (Fig. 2C).

Trajectories were grouped for analysis based on whether they included a time of collision (TOC), time of transition (TOT), or both (TOC/TOT). In trials where a subset of individual animals did not respond (i.e., no firing rate modulation) to a transition (i.e., H_T and 0_90_T), only responders were used in statistical analysis (*n* = 18 and 14, respectively). To characterize DCMD firing properties, we used similar methods to those described in McMillan and Gray (2012) and Silva et al. (2015). For all trajectories, we measured the total spike number during the entire stimulus presentation as well as the time leading up to transition. For all trajectories that contained a looming component (L_0, H_T, L_45, and 0_90_T), we quantified the positive peak firing rate (*f*_TOCp_) and peak time (*t*_TOC*p*_) associated with the projected TOC. We also quantified the negative peak (valley) firing rate (*f*_TOTv_) associated with TOT for trajectories that transitioned toward looming (H_T, 0_90_T) and the positive peak firing rate (*f*_TOTp_) associated with TOT for trajectories that transitioned away from looming (0_45, H_A, L_45_A, and 0_90_A). For transitions, we also measured the time of the respective valleys (*t*_TOTv_) or peaks (*t*_TOT*p*_ *)* relative to TOT. For trajectories H_A, H_T, and 0_90_T, TOT‐associated responses were masked by compression of the response duration in a flow field, and thus we were unable to discern a TOT‐associated valley or peak for this background. For trajectories with collision phases, we measured peak width of the peristimulus time histogram at ½ the maximal firing rate. The rise phase duration(s) of each DCMD response was calculated from the time at which the DCMD firing rate surpassed a 99% confidence interval (calculated from the entire stimulus presentation) to the time of peak DCMD firing rate (Fig. 2C). The TOC‐associated fall phase duration was designated as the time between when the stimulus stopped expanding and when the firing rate decreased to 15% of the peak DCMD firing rate (Gabbiani et al. 2005; Guest and Gray 2006). For trajectories that deviated from looming, TOT‐associated fall phases were designated as the duration from the maximum firing rate of the TOT‐associate peak to when the firing rate decreased to 15% of this value. For trajectories that deviated to looming, the maximum TOT‐associated firing rate occurred at TOT and thus the falling phase was equal to the delay (*δ*) from TOT to the time of the valley and, therefore, these data are equal to *t*_TOTv_ for trajectories H_T and 0_90_T. Previous studies describe how the rise and fall phases relate to stimulus‐evoked presynaptic network activity that drives LGMD/DCMD responses to motion (Gabbiani et al. 2002, 2005; Silva et al. 2015). For example, the fall phase relates to feed‐forward inhibition that terminates responses to looming (Gabbiani et al. 2005).

We also quantified object expansion parameters known to correlate with DCMD firing rate modulation (*f*′) and the delay from TOT to the time of the resulting peak or valley (*δ*): the instantaneous acceleration of: (1) the looming stimulus angular subtense (*θ*); and (2) the angular motion of the leading edge (*ψ*). Our data were pooled with those from McMillan and Gray (2012), Dick and Gray (2014) and Silva et al. (2015) and fit to unconstrained two‐dimensional Gaussian equations of the form:

(1) $$f^{\prime }=3.1e^{-0.5\left[{\left(\frac{\theta^{\prime \prime }+3.8}{33.3}\right)}^{2}+{\left(\frac{\psi^{\prime \prime }+166.8}{123.6}\right)}^{2}\right]}$$

(2) $$\delta =0.1e^{-0.5\left[{\left(\frac{\theta^{\prime \prime }+4.6}{38.5}\right)}^{2}+{\left(\frac{\psi^{\prime \prime }+352.2}{751.4}\right)}^{2}\right]}$$

where *θ*″ is the instantaneous acceleration of *θ*,* ψ*″ is the instantaneous acceleration of *ψ*, 3.1 and 0.1 represent the height of the mesh plot for *f*′ *and δ*, respectively, the numerical values within each successive numerator define the center of the peak of the mesh plot and the successive denominators relate to the width of the curve in the *x* and *y* planes, respectively.

### Statistical analysis

Firing parameters were tested for normality and equal variance in response to different trajectories and backgrounds with SigmaPlot 12.5 (Systat Software, Richmond, CA). DCMD firing parameters between initial and final frontal looms were compared using a *t*‐test for parametric data or a Mann–Whitney Rank Sum test for nonparametric data. The full data set did not satisfy tests for normality or equal variance. Therefore, we tested for significant differences across trajectories or backgrounds using a one‐way ANOVA on Ranks (reported by the *H* statistic). Flow fields resulted in no responses to a trajectory change for H_A and H_T and, therefore, data from simple and scattered backgrounds were compared using a Mann–Whitney Rank Sum test (reported by the *U* statistic). We used either a Tukey or Dunn's pairwise post hoc comparison for data with equal or unequal sample sizes, respectively. Table 1 summarizes the results of all statistical tests. All data were plotted as box plots showing the median value, 25th and 75th percentile as box boundaries, 10th and 90th percentiles as error bars, and outliers as small filled circles.

**Table 1:** Statistical comparison of measured DCMD response variables for all trajectories and backgrounds

Response variable	Trajectory								Background		
	L_0	0_45	H_T	H_A	L_45	L_45_A	0_90_T	0_90_A	Simple	Scattered	Flow
Figure 5											
# spikes	3.4_2_		16.9_2_ T		12.5_2_ T		11.6_2_ T		30.2_3_ D	13.0_3_ D	7.3_3_
*f*_TOCp_	11.4_2_ T		9.5_2_ T		0.9_2_		0.7_2_		18.5_3_ D	31.0_3_ D	36.7_3_D
PW½M	2.5_2_		27.5_2_ T		28.2_2_ T		8.1_2_ T		14.8_3_ D	9.4_3_ D	10.8_3_D
*t*_TOCp_	20.8_2_ D		35.2_2_ T		31.1_2_ T		4.4_2_		23.5_3_ D	4.4_3_	17.0_3_ D
Figure 6											
# spikes		38.8_2_ T	16.9_2_ T	27.2_2_T		35.4_2_ T	11.6_2_ T	26.5_2_ D	65.3_5_ D	51.4_5_ D	64.7_5_ D
*f*_TOTpv_		40.8_2_T	*U *=* *97	*U *=* *12		30.2_2_ D	*U *=* *69	25.7_2_ D	71.0_5_ D	38.3_5_ D	8.2_2_ D
*t*_TOTpv_		1.4_2_D	*U *=* *17	*U *=* *23		10.4_2_ D	*U *=* *82	18.3_2_ D	70.9_5_ D	43.1_5_ D	5.6_2_ D
Figure 7											
TOC_p_ rise	1.1_2_		5.4_2_		11.3_2_ T		7.5_2_ T		23.9_3_ D	1.4_3_	14.6_3_ D
TOC_p_ fall	21.1_2_ T		22.1_2_ T		14.0_2_ T		4.7_2_		6.4_3_	0.6_3_	2.0_3_
TOT_p_ rise		0.3_2_		*U *=* *6	4.2_2_			3.0_2_	16.6_3_ T	3.2_3_	4.8_2_
TOT_p_ fall		30.7_2_ D		*U *=* *3	0.6_2_			21.2_2_ D	33.7_3_ D	26.8_3_ D	2.0_2_

The columns under “Trajectory” report test results from comparisons across backgrounds within each trajectory. The columns under “Background” report test results from comparisons across trajectories within each background. Values represent the *H* statistic from one‐way ANOVA on Ranks test. Numerical subscripts indicate degrees of freedom. Post hoc tests denoted as Tukey (T) or Dunn's (D). Results from Mann–Whitney tests are indicated by the *U* statistic. Shaded cells indicate a significant effect (*P *<* *0.05). Specific differences from post hoc tests indicated in Figures 5, 6, 7. *f*_TOCp_, firing rate of TOC‐associated peak; *f*_TOTpv_, firing rate of TOT‐associated peak or valley; *t*_TOCp_, time of TOC‐associated peak; *t*_TOTpv_, time of TOT‐associated peak or valley; PW½M, peak width at half height.

## Results

The DCMD generated characteristic responses to frontal looms with an increasing firing rate that peaked before time of collision (Fig. 2C). We found no significant difference between the peak firing rate (*t*_*38*_ **= 0.437*)*, number of spikes (*U *=* *139.00), peak time (*t*_*38*_ **= −0.720), and peak width at ½ max (*U *=* *143.5) between initial and final frontal looms (data not shown), suggesting that DCMD responses were not affected by the duration of the experiment.

### Background motion affects TOC‐associated responses to object motion

**Figure 3:**
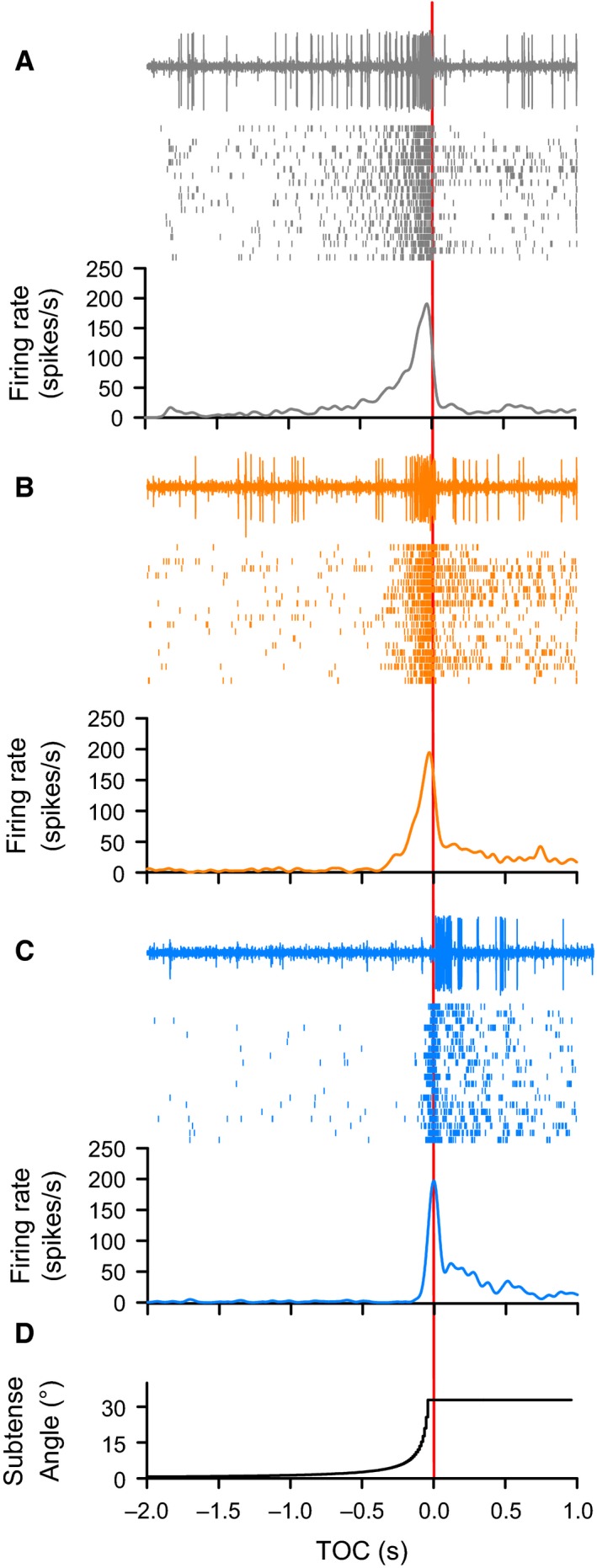
Peristimulus rasters for a looming stimulus approaching at a 45**°** angle relative to the eye (L_45). The top of each panel shows an individual raw recording. Large amplitude spikes of the DCMD were clearly distinguishable. Each middle panel shows the corresponding raster plot of spike times (black vertical lines) from each individual animal (*n* = 20) and each bottom panel shows the mean DCMD peristimulus time histogram. Response profiles are shown for the same trajectory presented in three visual backgrounds: (A) Simple (gray), (B) Scattered (orange), and (C) Flow Field (blue). Irrespective of background, the looming stimulus evoked a characteristic increase in firing rate. (D) The increasing visual subtense angle for a 7 cm black disk during a 45**°** loom. All panels time aligned to the projected time of collision (TOC, red vertical line).

**Figure 4:**
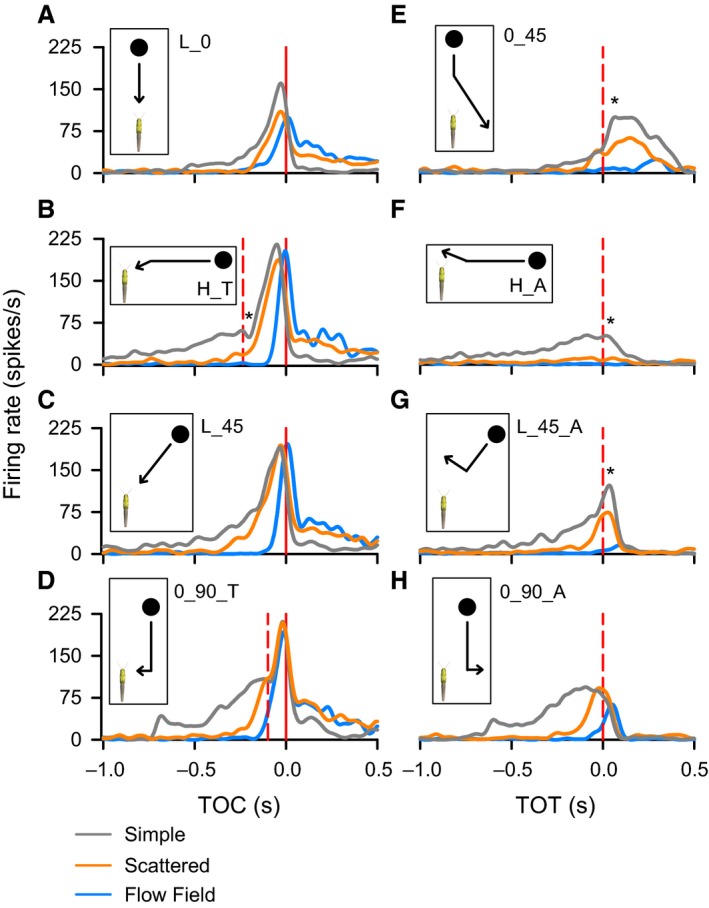
Mean DCMD responses to a 7 cm black disk travelling along eight unique trajectories against three different backgrounds. Peristimulus time histograms (PSTHs) of the DCMD show responses to the different categories of object motion (insets). Red solid vertical line in A–D represents TOC. The red dashed vertical line in B, D, E–H represents TOT, backgrounds represented by colored lines in the PSTHs: Simple (gray), Scattered (orange), Flow Field (blue). Asterisks indicate the time of a local valley (B) or peak (E, F, G,) in response to a transition.

**Figure 5:**
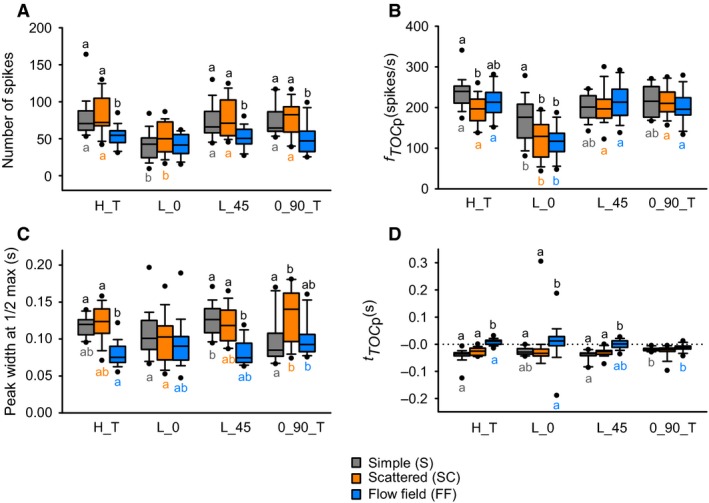
Effects of background and trajectory on TOC‐associated response variables. (A) Total number of spikes. (B) Peak firing rate. (C) Peak width at ½ maximum. (D) Peak time relative to TOC. Simple (gray), Scattered (orange), Flow Field (blue). Different letters above bars (black) represent significant differences of parameters between background type within each trajectory. Different letters below bars (color‐matched to bars) represent significant differences of parameters between trajectories within background type. Significance assessed at *P *<* *0.05. See Table 1 for statistical summary. *n* = 14–20 locusts.

We observed variable effects of background motion on qualitative aspects of the firing rate dynamics across all trajectories with a direct looming component, that is, objects approaching along a 45° trajectory (L_45, Figs 3, 4C) or a head on trajectory (L_0, Fig. 4A). Quantitatively, we found that for approaches at L_0, a simple background, resulted in a higher peak firing rate (Figs 4A and 5B) and a flow field induced a later peak (Figs 4A and 5D), whereas background motion had no effect on the number of spikes (Fig. 5A) or the peak width at half maximum (Fig. 5C). Background had no effect on the rise phase (Fig. 7A), whereas we observed a shorter fall phase for a simple background compared to either a scattered background or a flow field (Fig. 7B). For L_45, a flow field induced fewer spikes (Fig. 5A), a shorter peak width at half max (Fig. 5C), and a later peak (Figs 4C and 5D). We also observed no effect of trajectory on the rise phase (Fig. 7A) and a shorter fall phase for a simple background (Fig. 7B).

For compound trajectories, response parameters associated with TOC and TOT were analyzed separately if the trajectory contained both (i.e., H_T, 0_90_T). In addition to the two direct looming trajectories (L_0, L_45), trajectories containing both a TOT and TOC generated characteristic responses to the looming component of the stimulus (Fig. 4B,D).

For H_T, object motion resulted in fewer spikes in the presence of a flow field (Fig. 5A), a higher peak firing rate for a simple background (compared to a scattered background, Fig. 5B), a shorter peak width at half max for a flow field (Fig. 5C) and later peak firing rate for a flow field (Fig. 5D). While there was no effect of background on the rise phase (Fig. 7A), the fall phase was shorter for a simple background (Fig. 7B). For 0_90_T, a flow field resulted in fewer spikes during an approach (Fig. 5A) and a modest, though significantly shorter peak width at half max for a simple background compared to the presence of a scattered background (Fig. 5C). There was no effect of background on the amplitude (Fig. 5B) or time (Fig. 5D) of peak firing. The only effect on the rise phase was a slightly longer phase for 0_90_T for a simple background compared to a flow field. (Fig. 7A).

### Background motion affects TOT‐associated responses to object motion

**Figure 6:**
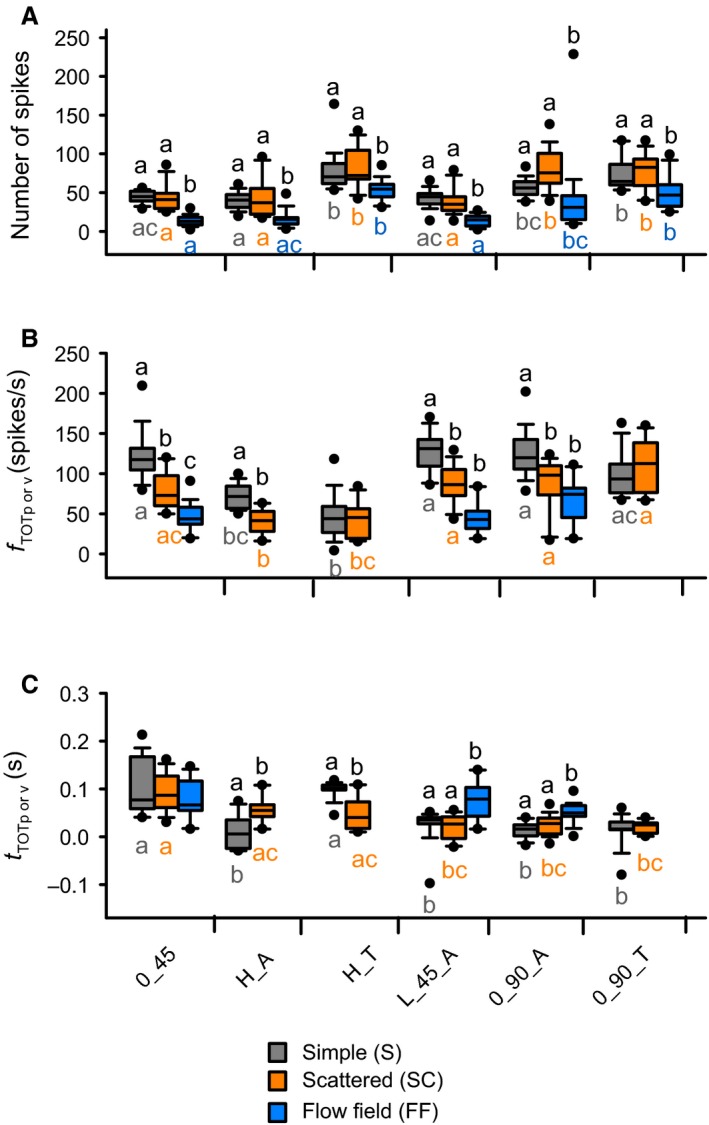
Effects of background and trajectory on TOT‐associated response variables. (A) Total number of spikes. (B) Peak or valley firing rate. (C) Peak or valley time relative to TOT. Box colors and indication of significant differences follow the same convention as Figure 5. Significance assessed at *P *<* *0.05. See Table 1 for statistical summary. *n* = 14–20 locusts.

We observed variable effects of background motion on firing rate dynamics across all compound trajectories with a transition toward or away from looming. Objects that transitioned toward looming included H_T and 0_90_T. For H_T, there were fewer spikes leading up to TOT in the presence of a flow field and an earlier TOT‐associated valley firing rate (shorter fall phase) in the presence of a scattered background (Fig. 6A and C, respectively). There was no effect of background on the valley firing rate (Fig. 6B). For 0_90_T, a flow field resulted in fewer spikes leading up to TOT (Fig. 6A), whereas there was no effect of background on the amplitude or time of the valley firing rate (Fig. 6B and C, respectively).

**Figure 7:**
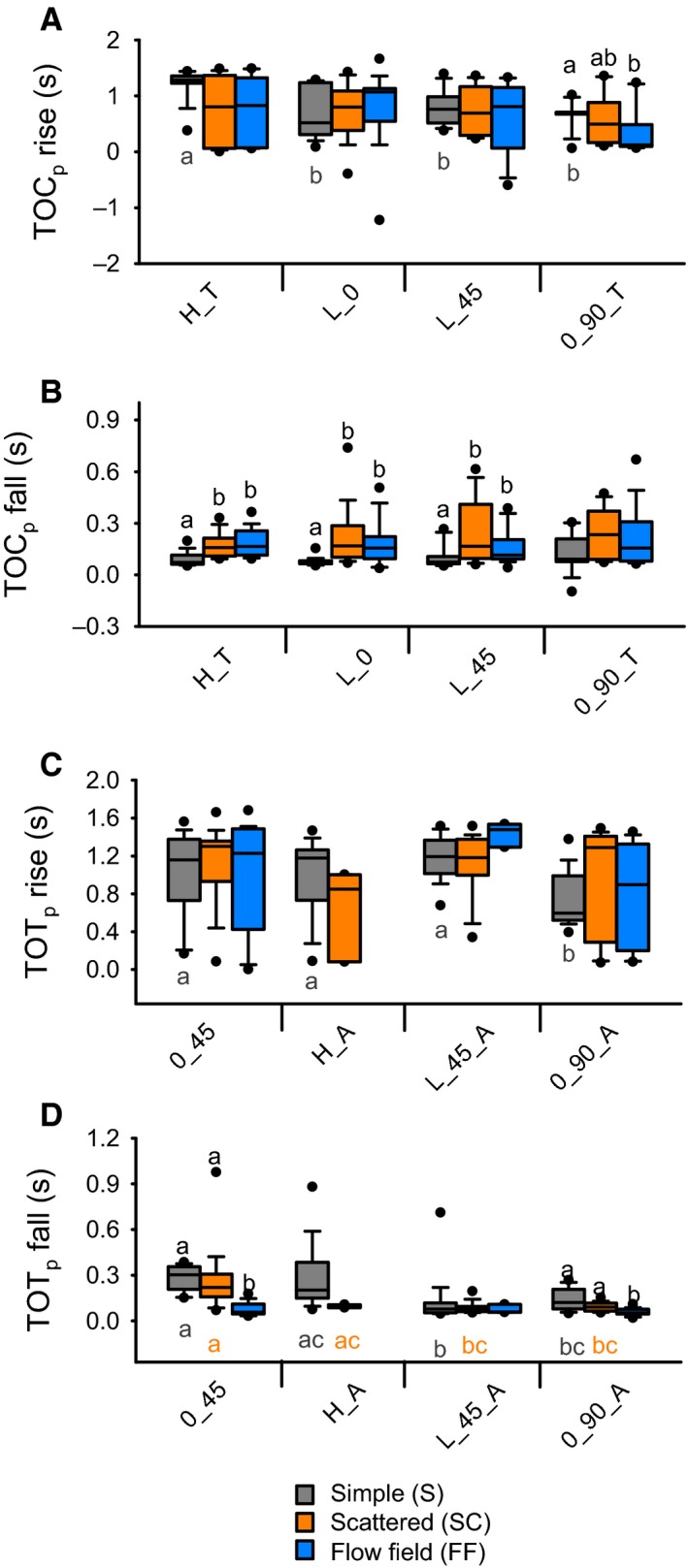
Effects of trajectory and background on TOT and TOC‐associated rise and fall phases (A) TOC peak rise phases were invariant to trajectory type and background. (B) TOC peak fall phases varied with background but not trajectory type. (C) TOT peak rise phases were relatively invariant to trajectory type and background. (D) TOT fall phases varied with trajectory type and background. Box colors and indication of significant differences follow the same convention as Figure 5. Significance assessed at *P *<* *0.05. See Table 1 for statistical summary. *n* = 14–20 locusts.

Transitions away from looming included 0_45, H_A, L_45_A, and 0_90_A. For 0_45, a flow field resulted in fewer spikes (Fig. 6A). We observed a progressively lower peak firing rate for simple, scattered, and flow field backgrounds (Fig. 6B), and there was no effect of background on the peak time (Fig. 6C) or the rise phase (Fig. 7C), whereas the fall phase was shorter in the presence of a flow field (Fig. 7D). For H_A, a flow field resulted in fewer spikes (Fig. 6A) and a scattered background evoked a lower peak firing rate (Fig. 6B) that occurred later (Fig. 6C). There was no effect of background on the rise or fall phases (Fig. 7C, D). For L_45_A and 0_90_A, a flow field resulted in fewer spikes and a later peak firing time (Fig. 6A and C, respectively), whereas a simple background resulted in a higher peak firing rate (Fig. 6C). The only effect of background on the rise or fall phases was a shorter fall phase in the presence of a flow field for 0_90_A (Fig. 7C, D).

### Object trajectory affects TOC‐associated responses to object motion

We observed clear qualitative differences in the DCMD response profiles between different object trajectories with a looming component (Fig. 4A–D). Quantitatively, a head on approach (L_0) resulted in fewer spikes for simple and scattered backgrounds, whereas there was no effect of trajectory in the presence of flow fields (Fig. 5A). L_0 also resulted in a lower peak firing rate relative to H_T for a simple background, a lower rate than all other trajectories for scattered and flow field backgrounds (Fig. 5B). The only differences we observed for peak width at half max was a longer duration for L_45 compared to L_0 and 0_90_T for a simple background, a longer duration for 0_90_T compared to L_0 for a scattered background, and a longer duration for 0_90_T compared to H_T for a flow field background (Fig. 5C). The time of peak firing occurred later for 0_90_T compared to H_T for a simple background and earlier than H_T and L_0 for a flow field (Fig. 5D). While the rise phase was longer for H_T compared to other trajectories for simple backgrounds (Fig. 7A), there was no effect of trajectory on the fall phase (Fig. 7B).

### Object trajectory affects TOT‐associated responses to object motion

Comparing the number of spikes leading up to TOT (Fig. 6A), we found that for objects approaching against a simple background, both H_T and 0_90_T evoked more spikes than 0_45, H_A and L_45_A and that 0_90_A evoked more spikes than H_A. For scattered backgrounds, H_T, 0‐90_A and 0_90_T evoked more spikes than the other trajectories. For flow fields, H_T and 0_90_T evoked more spikes than 0_45, H_A or L_45_A and 0_90_A evoked more spikes than 0_45 or L_45_A.

For a simple background, the TOT‐associated firing rate modulation was lower for H_A (peak) and H_T (valley), respectively. Against a scattered background, the TOT peak for H_A was lower than the responses for all other trajectories and the TOT valley for H_T was lower compared to L_45_A, 0_90_A, and 0_90_T. There was no effect of trajectory in the presence of a flow field (Fig. 6B).

The time of peak or valley firing (Fig. 6C), against a simple background was: later for 0_45 and H_T compared to other trajectories. For a scattered background, the peak or valley occurred later for 0_45 compared to L_45_A, 0_90_A, or 0_90_T. There was no effect of trajectory in the presence of a flow field (Fig. 6C).

The only effect of trajectory on the TOT‐associated rise phase for trajectories that deviated away from looming was shorter duration for 0_90_A (Fig. 7C). For fall phase (Fig. 7D) durations within simple backgrounds, 0_45 was longer than L_45_A and 0_90_A and H_A was longer than L_45_A. Against a scattered background the 0_45 fall phase was longer than for L_45_A and 0_90_A. There was no effect of trajectory on the fall phases in the presence of flow fields.

In summary, the data show that DCMD firing rate modulation varied with object trajectory and that flow fields generally evoked narrower and later TOC‐associated peaks without affecting the peak firing rate, except for head‐on approaches. The data also show that against a white background, DCMD firing rates transiently decreased for transitions toward looming and increased for transitions away from looming. These transition‐related modulations, however, were either mitigated or eliminated in the presence of background motion.

### Expansion properties at trajectory changes predict modulation of DCMD firing rate

**Figure 8:**
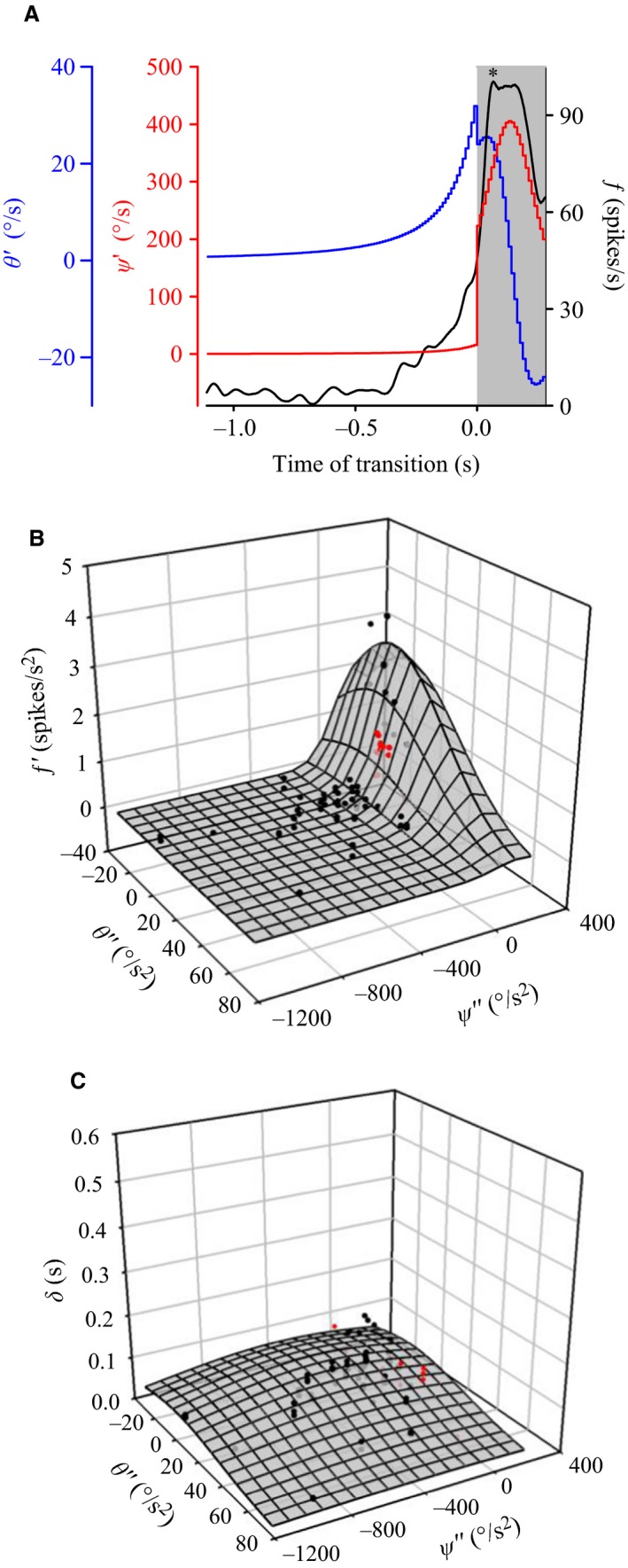
Correlation of DCMD firing modulation with object expansion parameters during transitions toward and away from looming. (A) Sample data illustrating the relationship between: an initial decrease in the object subtense angular velocity (*θ*′, blue line), increase in the velocity of the angular motion of the leading edge of the stimulus (*ψ*′*,* red line), and the resultant change in DCMD firing rate (*ƒ*, black line). Sample data in response to presentation of a disk along the 0_45 trajectory against a simple background. The gray shaded area indicates the duration when the stimulus travelled along a noncollision trajectory relative to TOT (*t* = 0 sec). *indicates a transient firing rate increase associated with a transition away from looming (see text). (B) Three‐dimensional scatterplot representing current data for objects transitioning toward or away from looming (red dots) plotted. Black dots represent pooled data from previous experiments (Dick and Gray 2014; McMillan and Gray 2012; Silva et al. 2015). All data were fit to an unconstrained two‐dimensional Gaussian equation (eq. (1), mesh plot, *r* ^2^ = 0.72). Data from the mean firing rate change (*ƒ*′) at TOT were plotted against the instantaneous object subtense angular acceleration (*θ*″) and the acceleration of the angular motion of the leading edge (*ψ*″) at the time of transition. (C) Plot of mean response time *(δ*) to instantaneous object subtense angular acceleration (*θ*″) and acceleration of the angular motion of the leading edge (*ψ*″). Data fit weakly to a Gaussian equation (eq. (2), mesh plot, *r* ^2^ = 0.18).

Previous experiments described the expansion parameters of objects that transition between translation and looming with different velocities and in the presence of different visual backgrounds (McMillan and Gray 2012; Dick and Gray 2014; Silva et al. 2015). Results from these studies demonstrate that transitions from looming resulted in a transient firing rate increase (*ƒ*′ > 1) that is correlated with unique expansion parameters of the disk. A decrease in firing rate (*ƒ*′ < 1) is correlated with an increase in the acceleration of the subtense angle (*θ*″) and an increase in the acceleration of the angular motion of the leading edge (*ψ*″). To confirm this relationship, our data were pooled with data from McMillan and Gray (2012), Dick and Gray (2014), and Silva et al. (2015). In addition to transitions to looming, our data added unique values of *θ*″ and *ψ*″ for trajectories that transitioned away from looming, which also resulted in an increase in mean firing rate (*ƒ*′ > 1, * in Fig. 8A). For the trajectories we used, transitions away from looming produced an increase in subtense angular acceleration *(θ*″* *> 0), except for L_45_A, which produced a decrease in object angular acceleration (*θ*″ < 0), and an increase in the acceleration of the angular motion of the leading edge (*ψ*″ *> *0). From equation (1), these expansion parameters correlated (*r* ^2^ = 0.72) with a pooled firing rate change, *ƒ*′, at the time of transition (Fig. 8B). When the data were separated by background and pooled with data from Silva et al. (2015), the fits for the data were: *r* ^2^ = 0.68, 0.76, and 0.72 for simple, scattered, and flow field, respectively (not shown). For response time *(δ*), data fit weakly to equation (2) (*r* ^2^ = 0.18) (Fig. 8C). Consistent with previous work, we confirm that *δ* remained invariant to stimulus trajectory and background.

## Discussion

We show that the complexity of visual backgrounds affects DCMD responses to moving objects that transition toward and away from a collision course. This is the first study to compare DCMD firing properties associated with objects that transition away from a collision course in the presence of background motion. We found that trajectory and background type had a significant effect on TOT‐ and TOC‐ associated firing properties. The scattered and flow field backgrounds affected peak firing time, spike numbers as well as dynamics associated with network properties that define peak firing, that is, the TOC‐associated peak fall phases. Our results support the hypothesis that the LGMD/DCMD pathway is able to convey information regarding the unique expansion parameters of a moving visual stimulus across variable trajectories and background complexity (McMillan and Gray 2012; Dick and Gray 2014; Silva et al. 2015).

The stimuli we selected here were designed to extend the range of object trajectories presented within previously defined backgrounds to further challenge the DCMD with unique object motion and transitions. These stimuli were also designed to generate values for *θ*″ and *ψ*″ that were unique from those we used previously in order to further test the robustness of our evolving correlational model on how this pathway responds to complex visual scenes. Nevertheless, our stimuli were similar to those from previous work that incorporated different trajectories or backgrounds (Judge and Rind 1997; Gabbiani et al. 2001; Gray et al. 2001; Guest and Gray 2006; McMillan and Gray 2012; Dick and Gray 2014; Silva et al. 2015) and thus allow us to place our findings within a broader context of DCMD coding of object motion.

### General responses to looming

Our data are consistent with other studies that show how the LCMD/DCMD pathway responds to looming with a characteristic increasing firing rate that peaks near the time of collision (Schlotterer 1977; Rind and Simmons 1992; Gabbiani et al.^1999^; Gray et al. 2001). While many studies provide evidence to explain biophysical mechanisms underlying network looming responses in this system (Gabbiani et al. 1999; Bermúdez i Badia et al. 2010; Meng et al. 2010; Yue and Rind 2013), we compare our work presented here to recent investigations into DCMD responses to changes in object trajectory and background motion complexity.

### Collision trajectories

For objects approaching in a direct collision course, our results are consistent with those from previous studies. For objects travelling head on toward the locust (0°), we observed a median peak firing rate of 176 spikes/sec (Fig. 5B) at median of 28 msec before collision (Fig. 5D), which is consistent with previous studies using comparable stimuli (Gray et al. 2001 – *l*/|*v*| = 17 msec; Silva et al. 2015 – *l*/|*v*| = 12 msec). Though they did not report a peak firing rate, Gabbiani et al. (2001) reported a peak time around 30 msec before collision for *l*/|*v*| = 10 msec. For objects approaching either directly from 45° azimuth (L_45) or with a trajectory component approaching from 45° (H_T), we observed statistically similar median peak firing rates of 201 (L_45) and 239 (H_T) spikes/s that occurred 34 msec (L_45) or 36 msec (H_T) before collision (see Fig. 5B and D). Using the same object properties, Guest and Gray (2006) reported a peak of approximately 200 spikes/sec around 5 msec before collision.

The design of our stimuli included trajectories with a component either offset parallel (0_90_T and 0_90_A) or perpendicular (H_T, H_A) to the longitudinal axis of the locust body. Our parallel offsets (30 cm before transitioning to looming) evoked lower maximum firing rates compared to those reported by Gray et al. (2001) for comparable stimuli. However, the stimuli used in Gray et al. (2001), did not pass through 90°. Rather, they stopped 10 or 20 cm from the locust (at a point 133 or 167 msec before reaching 90°) and were offset by 7 or 14 cm. Therefore, our data from our offset of 30 cm is consistent with their finding that a greater offset evoked a lower maximum firing rate (these rates cannot be defined as peaks as they did not pass through 90°).

Our offsets perpendicular to the locust's long axis differed from those used by Judge and Rind (1997). For their stimuli, the maximum offset was 16 cm anterior to the midpoint of the eye (equivalent to 90° in our coordinate system), whereas our offset was 50 cm (before transitioning to or from looming). Judge and Rind (1997) found no increase in firing rate during object motion when offset by more than 1.8° (6 cm). However, consistent with Silva et al. (2015), we did observe an increase in firing rate as the offset object approached the transition point. It is not clear if this difference is due to the object shape and slightly slower velocity used by Judge and Rind (1997; squares travelling at 2.5 m/sec).

### Transitions to looming

For our trajectories that transitioned to looming, the transition occurred 30 and 75 cm from the locust for 0_90_T and H_T, respectively. For 0_90_T across all backgrounds, we observed a median *f*_TOTv_ of 93–110 spikes/sec 16–25 msec after TOT whereas for H_T, *f*_TOTv_ was 44–45 spikes/sec 40–101 msec after TOT. Our 0_90_T and H_T are comparable to trajectories 2 and 1, respectively, from Silva et al. (2015) and, against a simple background, our 0_90_T is comparable to trajectories used by Dick and Gray (2014) and McMillan and Gray (2012). The differences from these previous studies are: (1) Silva et al. (2015) used slower object velocities (*l*/|*v*| = 40, 60 or 80 msec), whereas our *l*/|*v*| = 12 msec. Also, their transition for trajectory 1 occurred 50 cm from the locust, whereas our transition for H_T occurred 75 cm away; (2) Dick and Gray (2014) used a slightly faster approach velocity (*l*/|*v*| = 10 msec) and the transition occurred after translating from the rear, whereas our motion began from the front of the locust; and (3) McMillan and Gray (2012) used transitions that occurred 40 cm (for their A90‐40) or 80 cm (for their A90‐80) from the locust, whereas our transition occurred 30 cm away.

Across all trajectories, Silva et al. (2015) found that *f*_TOTv_ was affected by object velocity (generally decreasing with higher *l/|v|*) and background (decreasing with background complexity for *l/|v|* = 40 msec and 60 msec, but increasing with *l/|v|* = 80 msec). They also reported that *t*_TOTv_ was affected by velocity (increasing with //|v|) but was invariant to background complexity, occurring 69–115 msec after TOT. For our 0_90_T, we found no effect of background on *f*_TOTv_ or *t*_TOTv_. Across backgrounds, our values for *f*_TOTv_ and *t*_TOTv_ were 98–110 spikes/sec occurring 16–25 msec after TOT, which is greater and earlier than reported by Silva et al. (2015). For our H_T, while there was no effect of background on *f*_TOTv_, *t*_TOTv_ occurred earlier against a scatter background. Across backgrounds, our values for *f*_TOTv_ and *t*_TOTv_ were greater and earlier for a scattered background than reported by Silva et al. (2015) . Thus, our findings are consistent with an effect of object velocity on the amplitude and time of the valley. Our effect of background on *t*_TOTv_ for H_T and lack of effect of background for 0_90_T is likely due our faster object velocity (*l/|v *=* *12 msec), which would compress DCMD response durations overall and thus effect differences in valley amplitude and time. Nevertheless, our findings are consistent with a trend that *f*_TOTv_ decreases and occurs later after TOT as the transition distance from the locust increases (McMillan and Gray 2012; Dick and Gray 2014).

### Transitions from looming

Our transitions from looming (0_45, L_45_A, H_A, 0_90_A) were different from those of McMillan and Gray (2012); in that their objects transitioned from a trajectory that started at 90°, whereas ours started from 0°. Therefore, we cannot make direct comparisons of parameters associated with firing rate modulation following transition. Nevertheless, we did confirm an increase in firing following transitions away from looming and that the time of the response following TOT was relatively invariant to the location of the transition within the locust's field of view.

Estimates of conduction and processing delays for DCMD responses to objects looming from 0° or 90° range from approximately 2–45 msec (Gabbiani et al. 1999, 2001). These estimates are based on calculations of a delay between the object reaching a threshold subtense angle and the peak of DCMD firing. Our minimum response times following transition toward or away from looming are based on different, rapidly changing, stimulus parameters and ranged from the time of transition to 45 msec following transition. Response times below 2 msec may be due to other aspects of the complex stimuli as the object approaches the transition point. Nevertheless, the median values ranged from 6 to 100 msec following transition, suggesting that TOT‐ associated peaks or valleys represent responses to transitions. A more detailed comparison of conduction delays across a range of object approach velocities would address how processing upstream of the DCMD is affected by complex object motion.

### Implications for motion detection

Characteristic DCMD responses to looming, regardless of stimulus background, indicate that the locust remains sensitive to looming in the presence of complex visual information (see also Silva et al. 2015). While our flow field background may not fully replicate forward motion through three‐dimensional space, looming sensitive neurons are known to respond less robustly in the presence of optic flow (Simmons and Rind 1992; Gabbiani et al. 2002). Our findings show that a FF background generally resulted in fewer spikes for TOC and TOT‐associated trajectories. In some trajectories, the response to a transition was completely abolished in the presence of a progressive flow field. Specifically, objects that moved orthogonal to the direction of optic flow and transitioned away (H_A) or toward (H_T) evoked significantly fewer spikes and peak amplitude compared other backgrounds. Indeed, a flow field abolished TOT‐associated responses for H_A. Silva et al. (2015) found similar results and this effect has also been reported in pigeons (Xiao and Frost 2009). Flow fields also delayed a TOC‐associated peak firing rate for DCMD responding to objects on a collision course. Generally, peaks in the presence of a flow field occurred slightly after TOC, whereas against simple or scattered backgrounds, peaks occurred before TOC. This was also observed in the majority of trajectories that included transitions and can be attributed to a relatively longer duration of feed‐forward inhibition, which in turn would delay the accumulation of excitation (see discussion in Silva et al. 2015).

We observed a generally lower *f*_TOTp_, in the SC background than S for TOT trajectories, whereas *t*_TOCp_ was relatively invariant. Our scattered background attempts to emulate the presence of a swarm of conspecifics, where the visual pathway is exposed to a complex combination of visual stimuli with many regions of the visual field being stimulated simultaneously. Visual stimuli transitioning away could have been obscured due to the large number of additional visual objects within the locust's field of view in the scattered background. Our data confirm that the DCMD remained sensitive to approaches of looming objects in the presence of other object motion that could be attenuated by local habituation of presynaptic inputs to the LGMD (Gray 2005). Consistent with Silva et al. (2015), our results indicate that the combined properties of both object and background motion strongly affect DCMD response and that these components interact.

We found no effect of background on TOC or TOT‐associated peak rise phases. However, scattered backgrounds and flow fields did evoke longer TOC‐associated fall phases. In the presence of visual flow, Silva et al. (2015) also found a longer TOC‐associated fall phase, whereas Gabbiani et al. (2005) reported shorter rise phases, thus confirming that background motion affects detailed DCMD firing rate dynamics related to excitation and feed‐forward inhibition of LGMD by a presynaptic network (Gabbiani et al. 2002, 2005).

The stimulation and extinction of the DCMD response is largely affected by the kinematics of the stimuli (e.g., *l/|v|* values). Therefore, the differences in trajectory type also had an effect on the measured response parameters. For example, a relatively longer path of travel in closer proximity to the animal evoked more spikes (Fig. 6B). Moreover, complementary trajectories (transitioning from the same initial path: H_T and H_A, 0_90_T and 0_90_A) had the same overall response prior to TOT (Figs 5 and 6), which is expected given that the initial phase of stimulus presentation was identical.

### A correlational model for detecting complex object motion

The LGMD/DCMD pathway may be able to interpret information about the distinctive expansion properties of an object in motion irrespective of background complexity. Response time (*δ*) was variable (data not shown) among animals and trajectories, but consistent with previous studies (McMillan and Gray 2012; Dick and Gray 2014; Silva et al. 2015). Relative timing and amplitude modulation at the time of transition were affected by different rates of object expansion (*θ*″ and *ψ*″). Background motion has an effect on both unique expansion parameters and DCMD responses, but the correlation between such expansion parameters (*θ*″, *ψ*″) and the change in DCMD firing rate (*f*′) remains unaffected (Silva et al. 2015). McMillan and Gray (2012) first described these expansion parameters and fit changes in firing rate in response to transition changes in a 2D Gaussian equation. Here, we add our findings to earlier studies that varied object velocity (Dick and Gray 2014) and applied moving backgrounds to compound trajectories that transitioned toward the locust (Silva et al. 2015) to further support a correlational model.

Many animal groups also utilize looming sensitive neurons to detect salient stimuli. The praying mantis possess motion‐sensitive units that respond to looming in ways similar to that of the locust LGMD and may be involved in defensive behaviors (Yamawaki and Toh 2009; Sato and Yamawaki 2014). In the crab, *Chasmagnathus*, there are two subclasses of wide field movement detector neurons (MDNs) and MLG1 neurons are exceedingly sensitive to looming stimuli (Medan et al. 2007; Oliva et al. 2007). *Drosophila melanogaster* also possess looming sensitive neurons which are involved in initiating behavioral responses (Fotowat et al. 2009; De Vries and Clandinin 2012).The presence of looming sensitive neurons in other animal species may suggest that encoding of visual stimuli may occur in a similar way to the locust. Therefore, it is important to use backgrounds in combination with a moving disk to elucidate the importance of different aspects of complex stimulus environments. This study was unique in that it quantified DCMD responses to compound trajectories that transitioned away from the animal in the presence of a scattered background and in a progressive flow field. These types of stimuli are important in understanding how these neurons encode movement in a natural environment, which likely involves multiplexing of multiple sensory cues (Fotowat et al. 2011). As a result, our correlational model could provide a framework to test biophysical mechanisms of how visual systems may encode compound motion. Future work will examine if DCMD bursting properties related to looming detection (McMillan and Gray 2015) may play a role in responses to compound trajectories. Armed with detailed knowledge of DCMD responses to complex visual motion, it is now important to focus behavioral studies that examine flight steering within complex visual scenes. Also, given that the DCMD is not the only motion‐sensitive descending interneuron, it is important to make use of multichannel recording techniques to explore putative population‐level responses to more naturalistic scenes. Moreover, in the context of natural flight, it is possible that neuromodulation may be involved in the responses we observed here. Upregulation of octopamine during flight affects DCMD responses to looming, neural habituation, and the activity state of the locust during active maneuvering (Bacon et al. 1995; Rind et al. 2008). To gain a more complete understanding of collision detection and flight steering behavior, it would be valuable to incorporate measures of octopamine during presentation of complex scenes to a flying locust.

## Conflict of Interest

None declared.
